# Supplementary material for: Multiplexed detection of SARS-CoV-2 genomic and subgenomic RNA using in situ hybridization
Source: bioRxiv. 2021 Aug 11:2021.08.11.455959. Preprint. [Version 1] doi: 10.1101/2021.08.11.455959 (PMC8366794; doi:10.1101/2021.08.11.455959)
Supplement: 1 — Supplementary Figure 1: Single-molecule RNA FISH with probes targeting ORF1a and N regions in Huh7.5 cells infected with SARS-CoV-2. Representative images of Huh7.5 cells infected with SARS-CoV-2 and hybridized with RNA FISH probes targeting ORF1a and N. Similar to the RNA FISH HCR, we observed higher fluorescence signal intensity in the perinuclear region of the ORF1a probe compared to the N probe. The bottom row of images is a composite of the ORF1a probe (green), N probe (red), and DAPI signal. In all images, the DAPI stain for cell nuclei is shown in blue. Scale bars are 10 μm. Supplementary Figure 2: Examples of regions in the lung tissue that show viral staining with ORF1a probes, but do not have nuclei. A. Two example regions in which we observed extensive viral staining with the ORF1a probe set, but did not observe DAPI signal. Right image is RNA FISH HCR for ORF1a and the left is the brightfield image. Red dotted lines show areas of interest with ORF1a staining. B. Examples of small discrete regions of ORF1a staining without DAPI staining. In all images, the DAPI stain for cell nuclei is shown in blue. Scale bars are 10 μm. Supplementary Figure 3: Computational analysis identifying suspected infected cells in each tissue. Images overlayed with the results from our image processing pipeline. Blue dots label cells with fluorescence signal below the cutoff for infected and red dots label cells above the cutoff for infected. Images are from a subset of the data. Total number of cells displayed in each region and the total number of cells in each data set is below each image. Supplementary Figure 4: Comparison of infected tissue to control tissues samples in which the patients did not have SARS-CoV-2 infection. We performed RNA FISH HCR in A. lung and B. placenta samples from patients with SARS-CoV-2 infection (yellow) and control samples from patients that did not have SARS-CoV-2 infection (purple and teal). Histograms display the Log2 transformation of median [file NIHPP2021.08.11.455959V1-supplement-1.pdf]

bioRxiv preprint doi: https://doi.org/10.1101/2021.08.11.455959; this version posted August 11, 2021. The copyright holder for this preprint (which was not certified by peer review) is the author/funder, who has granted bioRxiv a license to display the preprint in perpetuity. It is made available under aCC-BY 4.0 International license.

Supplementary Figure 1

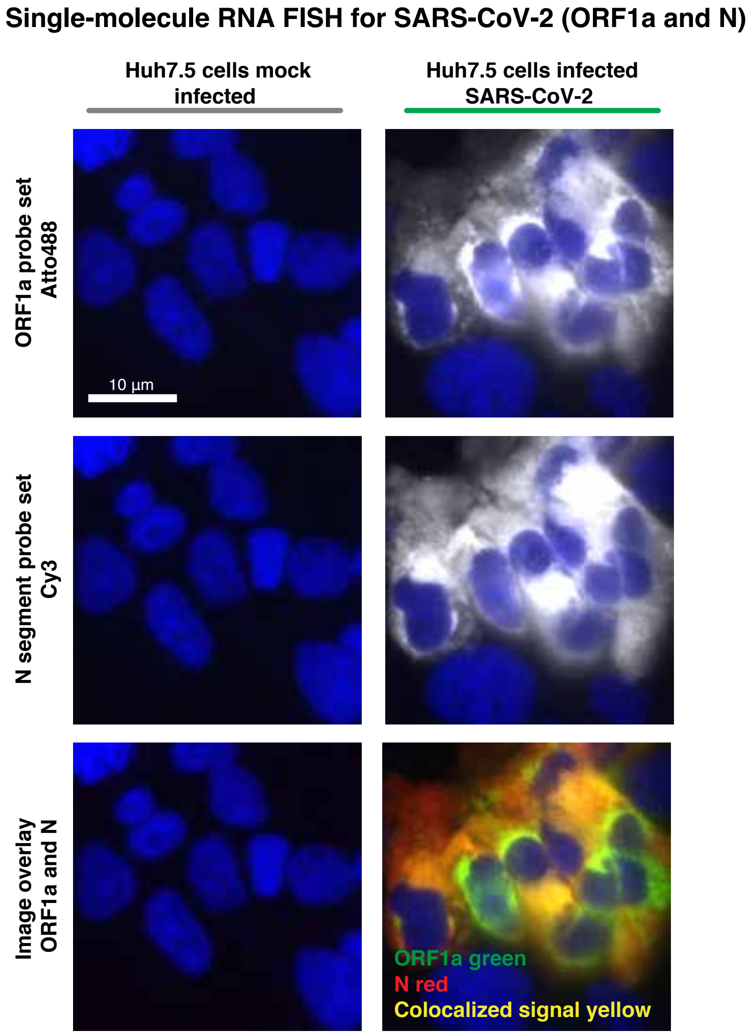

Supplementary Figure 1: Single-molecule RNA FISH with probes targeting ORF1a and N regions in Huh7.5 cells infected with SARS-CoV-2. Representative images of Huh7.5 cells infected with SARS-CoV-2 and hybridized with RNA FISH probes targeting ORF1a and N. Similar to the RNA FISH HCR, we observed higher fluorescence signal intensity in the perinuclear region of the ORF1a probe compared to the N probe. The bottom row of images is a composite of the ORF1a probe (green), N probe (red), and DAPI signal. In all images, the DAPI stain for cell nuclei is shown in blue. Scale bars are 10 µm.

bioRxiv preprint doi: https://doi.org/10.1101/2021.08.11.455959; this version posted August 11, 2021. The copyright holder for this preprint (which was not certified by peer review) is the author/funder, who has granted bioRxiv a license to display the preprint in perpetuity. It is made available under aCC-BY 4.0 International license.

Supplementary Figure 2

a **Regions of viral staining without nuclei**

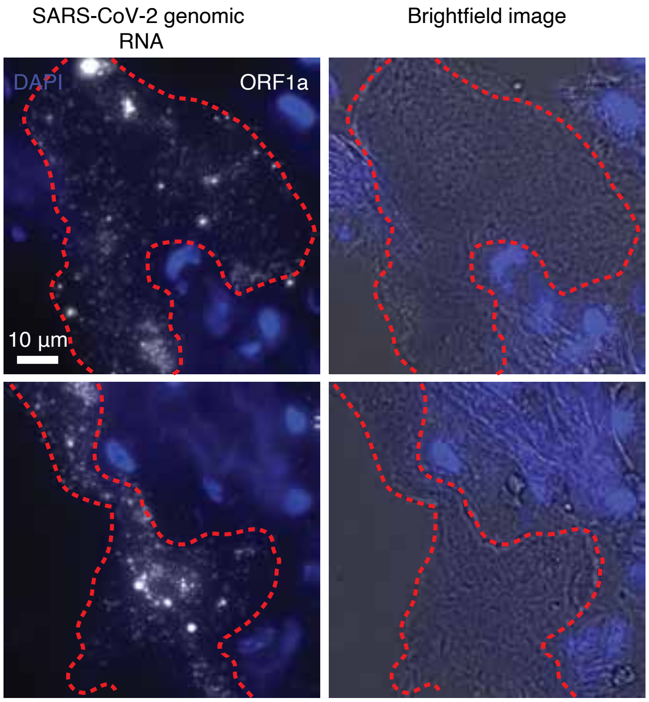

b **Examples of smaller discrete regions of viral RNA without nuclei**

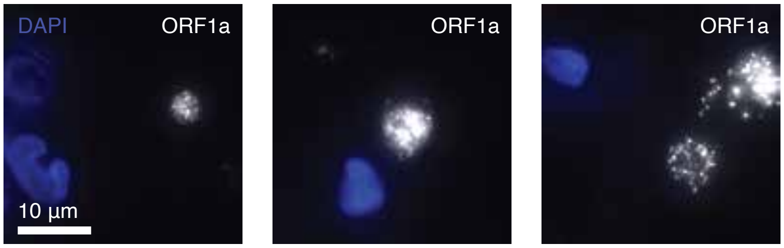

Supplementary Figure 2: Examples of regions in the lung tissue that show viral staining with ORF1a probes, but do not have nuclei. A. Two example regions in which we observed extensive viral staining with the ORF1a probe set, but did not observe DAPI signal. Right image is RNA FISH HCR for ORF1a and the left is the brightfield image. Red dotted lines show areas of interest with ORF1a staining. B. Examples of small discrete regions of ORF1a staining without DAPI staining. In all images, the DAPI stain for cell nuclei is shown in blue. Scale bars are 10 µm.

bioRxiv preprint doi: https://doi.org/10.1101/2021.08.11.455959; this version posted August 11, 2021. The copyright holder for this preprint (which was not certified by peer review) is the author/funder, who has granted bioRxiv a license to display the preprint in perpetuity. It is made available under aCC-BY 4.0 International license.

Supplementary Figure 3

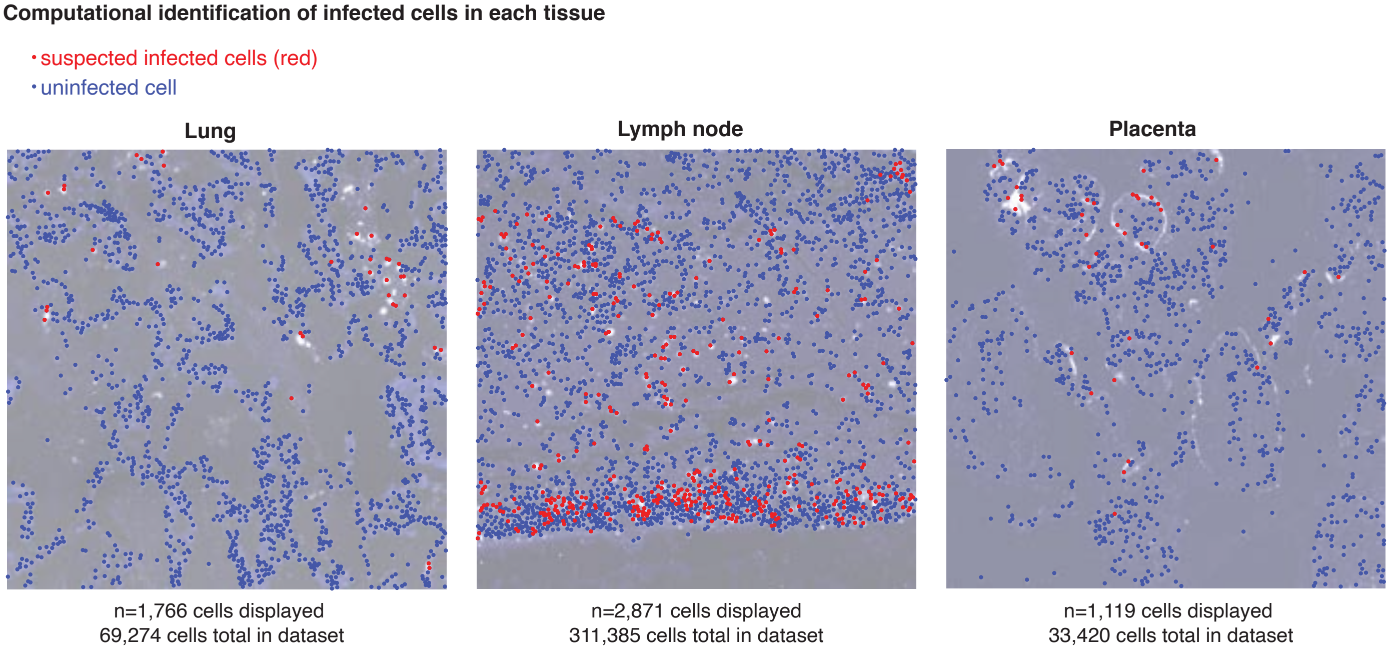

Supplementary Figure 3: Computational analysis identifying suspected infected cells in each tissue. Images overlayed with the results from our image processing pipeline. Blue dots label cells with fluorescence signal below the cutoff for infected and red dots label cells above the cutoff for infected. Images are from a subset of the data. Total number of cells displayed in each region and the total number of cells in each data set is below each image.

bioRxiv preprint doi: https://doi.org/10.1101/2021.08.11.455959; this version posted August 11, 2021. The copyright holder for this preprint (which was not certified by peer review) is the author/funder, who has granted bioRxiv a license to display the preprint in perpetuity. It is made available under aCC-BY 4.0 International license.

Supplementary Figure 4

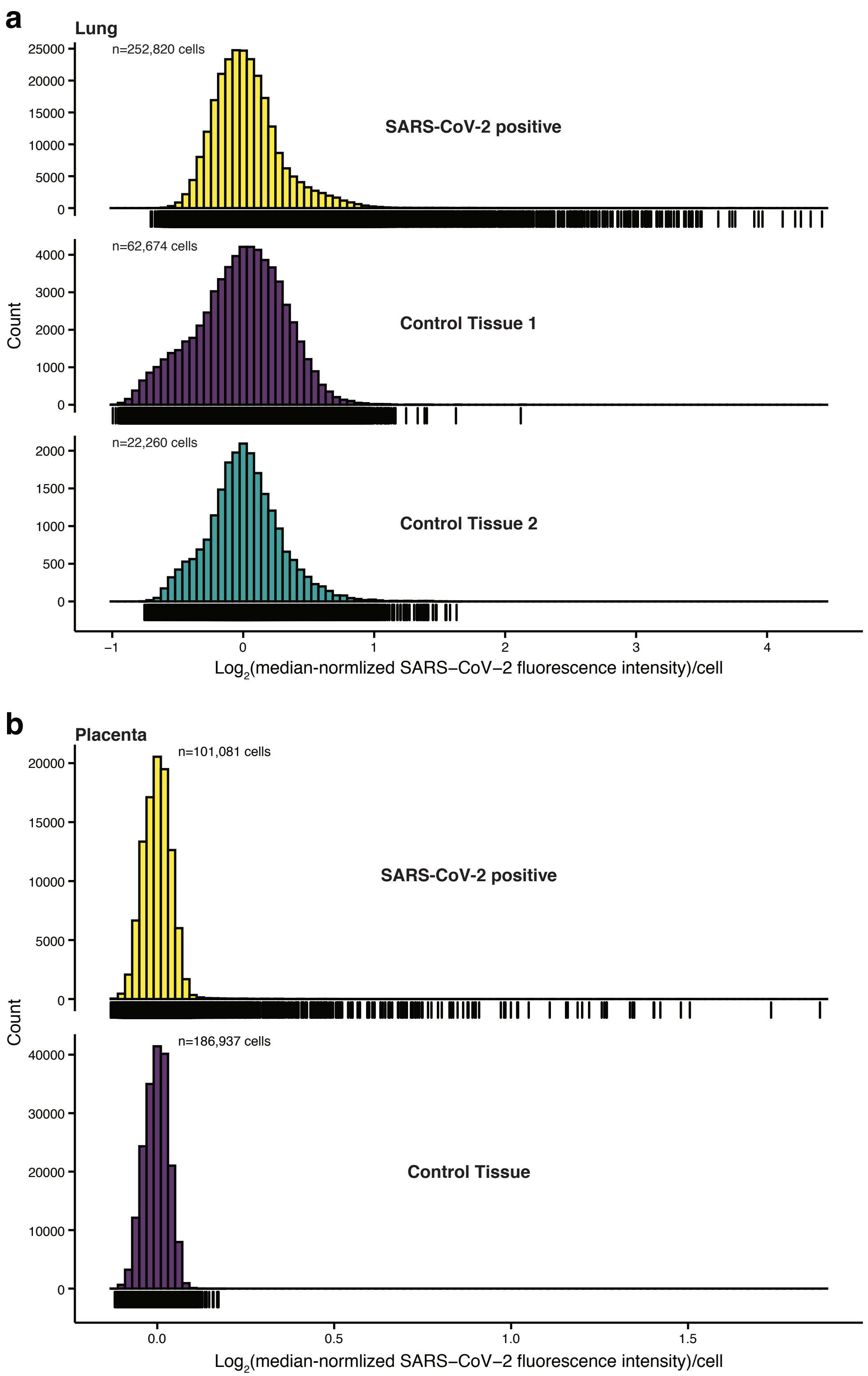

Supplementary Figure 4: Comparison of infected tissue to control tissues samples in which the patients did not have SARS-CoV-2 infection. We performed RNA FISH HCR in A. lung and B. placenta samples from patients with SARS-CoV-2 infection (yellow) and control samples from patients that did not have SARS-CoV-2 infection (purple and teal). Histograms display the Log2 transformation of median normalized ORF1a fluorescence signal in each cell. Infected samples have much higher median-normalized ORF1a fluorescence signal than control tissue.

bioRxiv preprint doi: https://doi.org/10.1101/2021.08.11.455959; this version posted August 11, 2021. The copyright holder for this preprint (which was not certified by peer review) is the author/funder, who has granted bioRxiv a license to display the preprint in perpetuity. It is made available under aCC-BY 4.0 International license.

Supplementary Figure 5

Placenta ORF1a RNA staining **H&E adjacent slide**

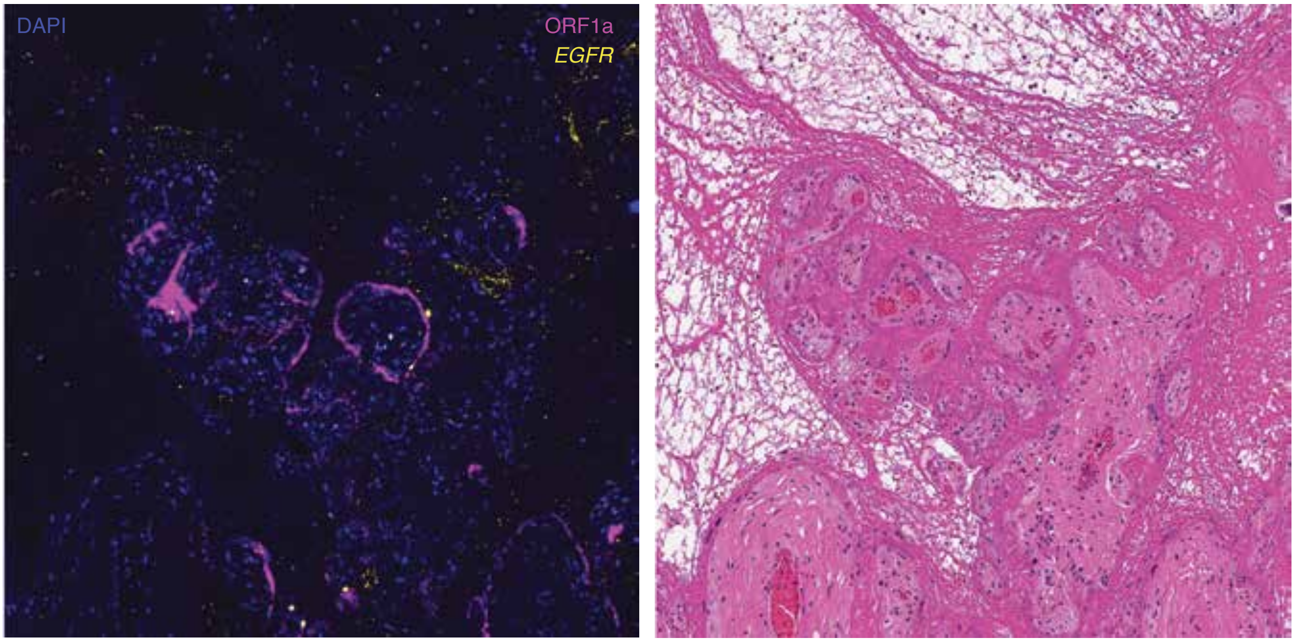

Supplementary Figure 5: Example region of placenta with RNA FISH HCR for ORF1a with an adjacent tissue section stained with H&E. We performed RNA FISH HCR with probe sets for ORF1a and *EGFR*. On the adjacent section, we stained the tissue with hematoxylin and eosin (H&E). We took tiled image scans of the fluorescence slide and used a slide scanner for the H&E. We aligned the two images to identify the corresponding H&E region for which we found cells staining with the ORF1a probe set. ORF1a fluorescence signal is in pink, *EGFR* is in yellow, and DAPI is in blue.

bioRxiv preprint doi: https://doi.org/10.1101/2021.08.11.455959; this version posted August 11, 2021. The copyright holder for this preprint (which was not certified by peer review) is the author/funder, who has granted bioRxiv a license to display the preprint in perpetuity. It is made available under aCC-BY 4.0 International license.

Supplementary Figure 6

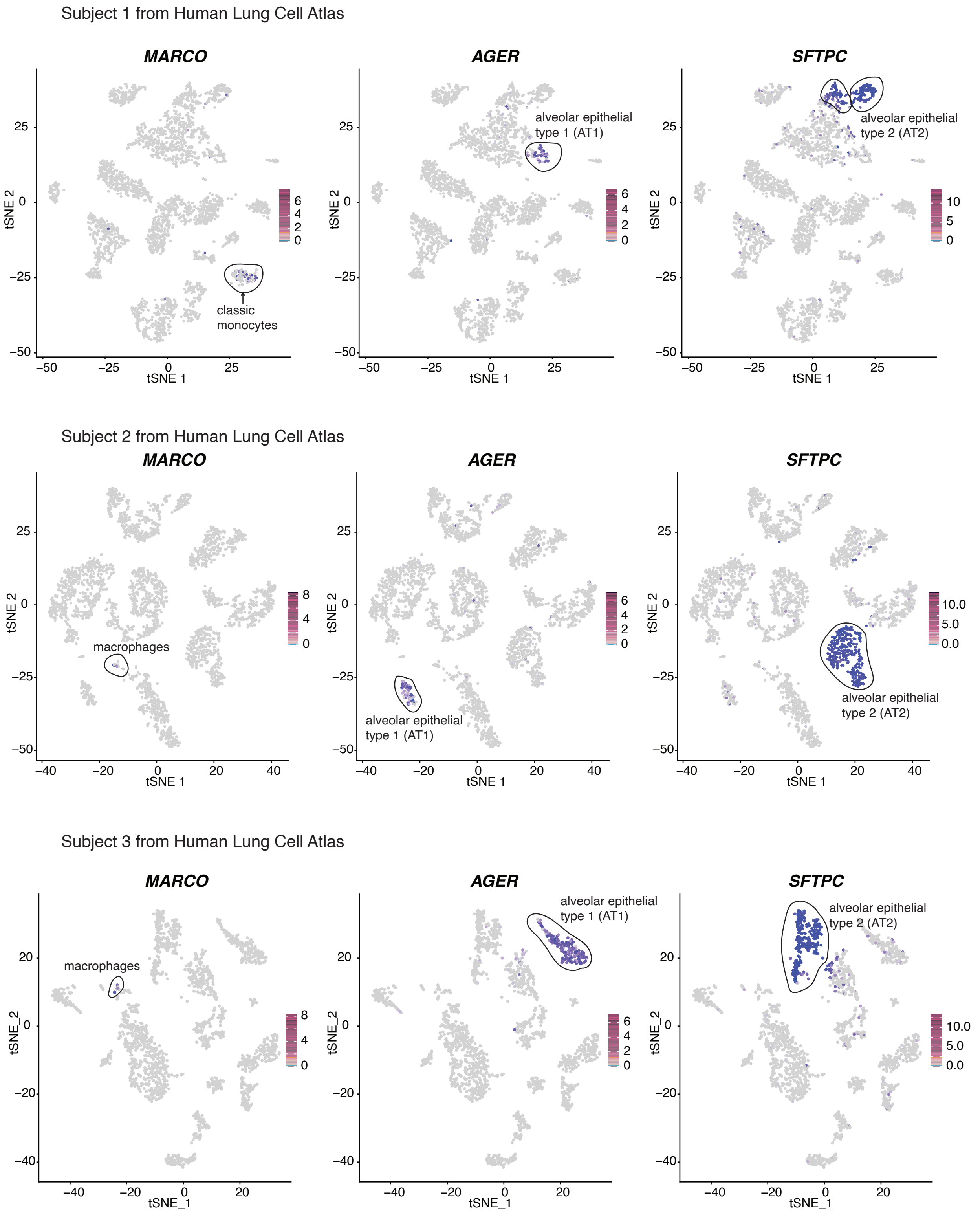

Supplementary Figure 6: tSNE plots of the human lung cell atlas single-cell RNA-sequencing data across 3 subjects. Each plot is a tSNE projection of all cells in the data set with the color of the points depicting the expression of the gene. Each row of plots is from a different subject. The target cell-type with each marker is labeled on the plots with a circle around the cluster (monocytes/macrophages, AT1 cells, AT2 cells). The three genes identified as cell-type specific markers are *MARCO*, *AGER*, and *SFTPC*.
